# Supplementary material for: Clinical Impact of Supplementation with Pasteurized Donor Human Milk by High-Temperature Short-Time Method versus Holder Method in Extremely Low Birth Weight Infants: A Multicentre Randomized Controlled Trial
Source: Nutrients. 2024 Apr 8;16(7):1090. doi: 10.3390/nu16071090 (PMC11013736; doi:10.3390/nu16071090)
Supplement: Supplementary file 1 [file nutrients-16-01090-s001.zip › nutrients-2939317-supplementary/Supplementary Tables.pdf]

SUPPLEMENTARY MATERIALS

Table S1. Clinical variables during admission considering the 2 groups of pasteurization.

	HTST GROUP (N=79)	HOLDER GROUP (N=81)	P value
	**Median (IQR)**	**Median (IQR)**	
Catheter days	16 (10; 26)	18 (10;32)	0.29
Days of parenteral nutrition (admission)	18 (11;27)	21 (10;35)	0.33
Days of antibiotics during (admission)	9 (2;20)	9 (3;23)	0.36
Days of antibiotics first 28 days of life	7 (3;13)	8 (2;17)	0.64
Days of probiotics during admission	12 (0;43)	20 (0;39)	0.69
DHM volume first 28 days of life (mL/kg/day)	5.2 (1.8;22.5)	6.8 (2.1; 32)	0.3
MOM volume first 28 days of life (mL/kg/day)	56.6 (25.8; 94.2)	35.3 (8.9; 77.9)	**0.017**
DHM volume up to 34 weeks PMA (mL/kg/day)	5.9 (1.4;53.8)	25.8 (4.2; 56.3)	0.14
MOM volume up to 34 weeks PMA (mL/kg/day)	84,6 (3.4;118.2)	48 (7.4; 107.8)	**0.015**
Nº doses oropharyngeal colostrum first 4 days of life	3 (0;9)	3 (0;7)	0.33
	**N (%)**	**N (%)**	
Postnatal corticosteroids	12 (15)	13 (16.1)	0.88
Received anti-acids (admission)	24 (30.4)	17 (21)	0.17
Received vasoactive drugs (admission)	32 (41)	34 (42)	0.65
Received ibuprofen for PDA (admission)	35% (28)	25 (31)	0.52
Received paracetamol for PDA (admission)	27% (21)	20 (25)	0.77

IQR: interquartile range; DHM: donor human milk; MOM: mother's own milk; PMA: postmenstrual age; PDA: patent ductus arteriosus.

Table S2 Baseline characteristics by study center.

	CENTER 1	CENTER 2	
	N=95	N=65	
	N (%)	N (%)	P value
Sex (Male)	44 (46)	25 (38)	0.32
Multiple pregnancy (≥2)	33 (34.7)	20 (30.8)	0.36
Pregnancy (Well-controlled)	89 (93.7)	57 (87.7)	0.19
Country of origin Spain	54 (58)	39 (67)	0.26
Antenatal corticosteroids (≥ 1 full course)	80 (84.2)	47 (72.3)	0.07
Low weight for gestational age (p < 10)	34 (36)	12 (18)	**0.017**
	**Median (IQR)**	**Median (IQR)**	**P value**
Gestational age (weeks)	27 (25; 28)	26 (25;27)	0.12
Birth weight (grams)	820 (640; 920)	817 (716; 897)	0.49
Fenton z score for birth weight	-0.7 (-1.5;-0.2)	-0.2 (-1;+0.2)	**0.012**
Apgar score 5 minutes	8 (6;9)	7 (5;5.9)	0.078
CRIB I score	7 (2;9)	5 (2;8)	0.34

IQR: interquartile range; CRIB: index of clinical risk in babies, first version.

Table S3 Clinical variables during admission by study center.

IQR: interquartile range; DHM: donor human milk; MOM: mother's own milk; PMA: postmenstrual age; PDA: patent ductus arteriosus.

	CENTER 1 (N=95)	CENTER 2 (N=65)	P value
	**Median (IQR)**	**Median (IQR)**	
Catheter days	17 (10;31)	18 (10;27)	0.636
Days of parenteral nutrition (admission)	18 (11;33)	20 (11;28)	0.73
Days of antibiotics during (admission)	7 (2;18)	16 (6;27)	0.056
Days of antibiotics first 28 days of life	5 (2;10)	12 (5;19)	**<0.001**
Days of probiotics during admission	39 (21;48)	0 (0;0)	**<0.001**
DHM volume first 28 days of life (mL/kg/day)	8.7 (2.8;35.9)	3.4 (1.4;16.1)	**0.004**
MOM volume first 28 of life (mL/kg/day)	43.2 (12.9; 89.2)	54 (22;81.2)	0.69
DHM volume up to 34 weeks PMA (mL/kg/day)	15.2 (4.3;69.8)	3.7 (1.2;40.8)	**0.03**
MOM volume up to 34 weeks PMA (mL/kg/day)	54.6 (12.4;112.6)	81.6 (17.3;109.7)	0.45
Nº doses oropharyngeal colostrum first 4 days of life	3 (0;9)	3 (0; 7)	0.33
	**N (%)**	**N (%)**	
Postnatal corticosteroids	12 (12.6)	13 (20)	0.20
Received probiotics (admission)	92 (97)	0 (0)	**<0.001**
Received anti-acids (admission)	7 (7.4)	34 (52.3)	**<0.001**
Received vasoactive drugs (admission)	37 (39)	29 (45)	0.39
Received ibuprofen for PDA, (admission)	30 (32)	23 (35)	0.49
Received paracetamol for PDA (admission)	16 (17)	25 (38)	**0.002**
