# Supplementary figures and images for: Clinical Impact of Supplementation with Pasteurized Donor Human Milk by High-Temperature Short-Time Method versus Holder Method in Extremely Low Birth Weight Infants: A Multicentre Randomized Controlled Trial
Source: Nutrients. 2024 Apr 8;16(7):1090. doi: 10.3390/nu16071090 (PMC11013736; doi:10.3390/nu16071090)

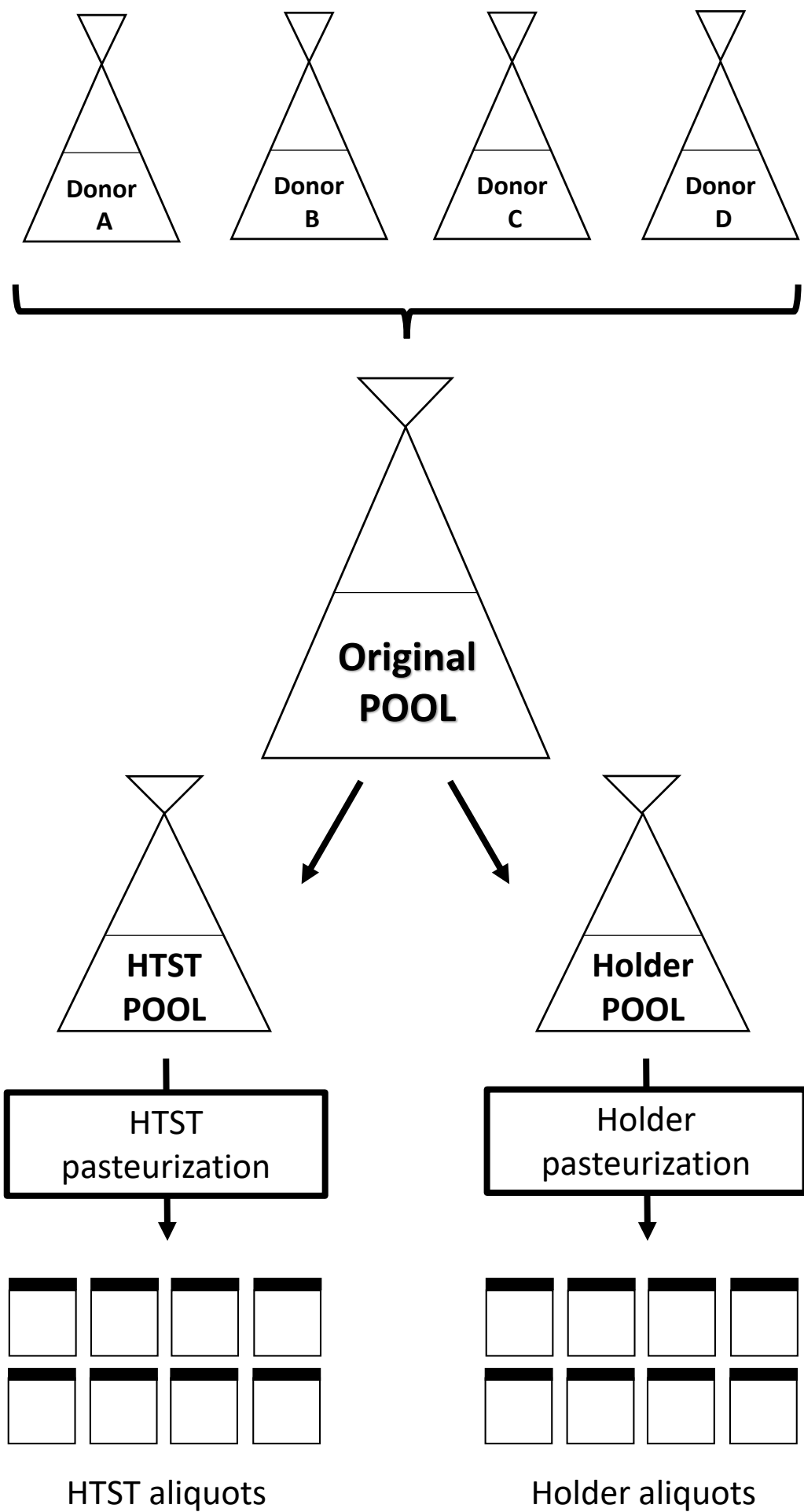

**Figure S1. DHM batches for study**

Supplement: Supplementary file 1 [file nutrients-16-01090-s001.zip › nutrients-2939317-supplementary/FIGURE S1. DHM batches for study.pdf]

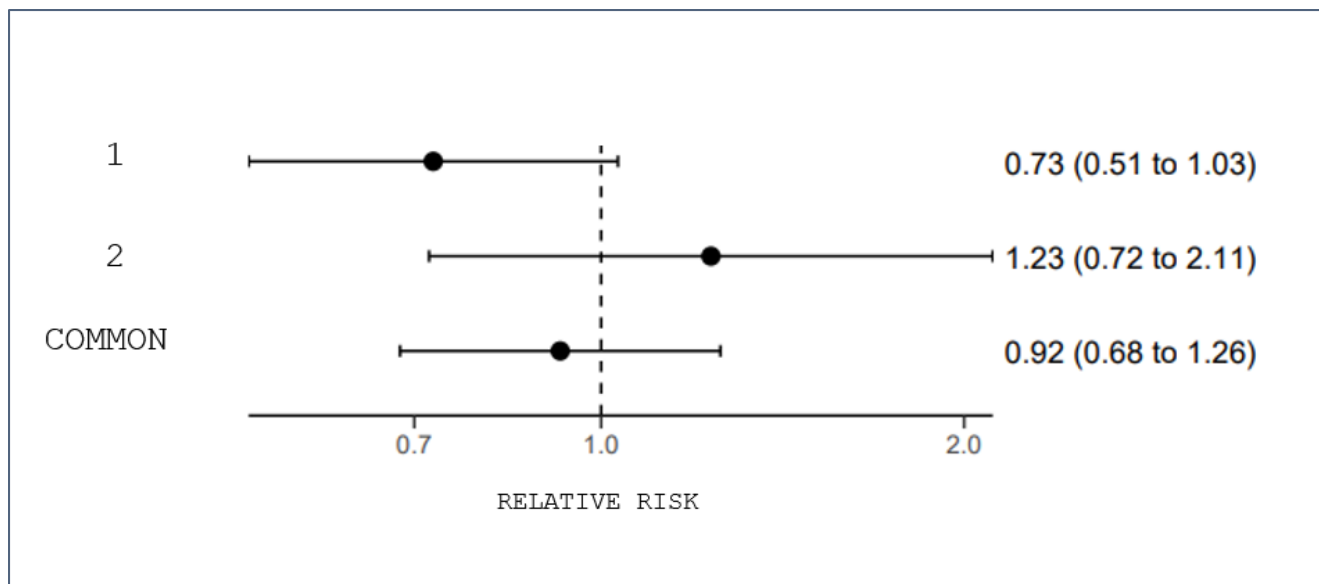

Figure S2. Subgroup effect (HTST vs Holder) by study centre (Relative risk and 95% CI)

Supplement: Supplementary file 1 [file nutrients-16-01090-s001.zip › nutrients-2939317-supplementary/FIGURE S2. Main outcome. RR by study site.pdf]
